# A systematic literature review on cybercrime legislation

**DOI:** 10.12688/f1000research.123098.1

**Published:** 2022-08-23

**Authors:** Shereen Khan, Tajneen Saleh, Magiswary Dorasamy, Nasreen Khan, Olivia Tan Swee Leng, Rossanne Gale Vergara

**Affiliations:** 1 Faculty of Management, Multimedia University, Cyberjaya, Selangor, 63100, Malaysia; 2 Faculty of Business and Law, Taylor's University, No. 1, Jalan Taylors, 47500 Subang Jaya, Malaysia

**Keywords:** Cybercrime, Legislation, Cybersecurity, Cyber legislation, Online crime, Systematic literature review, Combating, Legal Framework, Online Fraud

## Abstract

**Background**: Cybercrime is a fast-growing digital crime and legislation falling behind with the fast-moving advancement of technology. One important factor projected by literature in combating cybercrime is legislation. In order to combat cybercrime, the role of cybercrime legislation is a challenge that has not been clearly studied before. This paper thus aims to recapitulate the literature on cybercrime legislation in combating cybercrime. The literature in this context emphasises on existing studies relating to cybercrime legislation and addressing the importance of adequate and efficient responses in place in order to combat cybercrime efficiently.

**Methods**: This paper finds an extensive literature review using the “Preferred Reporting Items for Systematic Review and Meta-Analysis” method based on legislation to combat cybercrime and explains a systematic analysis of the legislation in most advanced countries in both technology and legal framework. The study was done by selecting keywords, validated by the experts to discover research trends of cybercrime legislation. A search was then run across seven academic databases, including the ACM Digital Library, Emerald, Hein Online, ProQuest, ScienceDirect, Scopus, and Westlaw Asia. Initially, five hundred and forty-eight articles were retrieved and out of which seventy-two studies met the inclusion criteria and were fully reviewed.

**Results**: The findings of the study revealed that comprehensive cybercrime legislation plays a vital role in combating cybercrime and cybercrime legislation should be strengthened, enhanced, and made up to date with the rapid advancement of technology in order to address the rising number of cybercrime.

**Discussion**: This systematic review is timely and highlights future research directions to improve a comprehensive legal framework to combat the rising of cybercrime effectively. To fill the research gap, the findings also have fundamental practical implications for the policy makers, in enacting an up to date cybercrime legislation by highlighting the role of legislation in combating cybercrime.

## Introduction

The year 2020 had been an unprecedented challenging year especially from cybercrime perspectives. Not only that the world has to face the threats from global pandemic outbreak, but also from the increasingly sophisticated cyber-attacks. Global pandemic outbreak has resulted in more than 600% rise in cybercrime. ^1^ The more sophisticated and advanced the technology is, the higher the number of cybercrimes and the more difficult to comprehend the issue. Cybercriminals use the advanced technology to do criminal activities such as hacking, phishing, spamming, and child pornography and hate crimes resulting in massive losses suffered by individuals as well as corporations and even pose danger to national security in some cases.

According to the report by Reuters on 5 ^th^ July 2021, when the information technology firm from the United States was attacked by cybercriminals, around 800 to 1,500 businesses were affected around the world. It was reported by the FBI that 791,790 cybercrime complaints resulting in more than US$4.1 billion losses were received by the Internet Crime Complaint Centre in 2020 and that number of reported cases has increased 69 percent compared to the reported cases in 2019. It is not only that there is an increase in cybercrime cases, the level of sophistication of the technology used also has increased tremendously. The 2020 “SolarWinds” attack confirmed the reality of the sophistication of the technology involved. “SolarWinds” attack in 2020 was by the state sponsored attack since Russian military hackers accessed and sabotaged the United States Government databases and buried a software called SolarWinds. The latest attack being the largest online theft reported by CNN on the 12 ^th^ August was about the hackers who had stolen US$600million worth of cryptocurrencies from the decentralised finance platform of Poly Network. It shows how sophisticated the technology has become and how weak the legislation is to comprehend and deter these attacks.

Cybercrime can be categorised as crimes exclusive to internet and use of computer and traditional crimes. Crimes such as stalking, harassing, fraud or scam committed using social media such as Facebook, Instagram, Twitter can be regarded as traditional crimes which are committed entirely in new ways. Criminal acts done by using electronic communications and information systems are generally considered as cybercrime which consists of a variety of criminal acts. It can be individual acts as well as state sponsored cybercrime. There is no definition of cybercrime which has been accepted globally. However, the term cybercrime has been used to describe a range of crimes excluding traditional crimes but crimes committed using the computer network system. Because of the complexity of the nature of crime, a single act of cybercrime can cause overly high damages. Cybercrime currently contributes to the highest percentage of all crime. Historically, legislation has been used as a way to combat the challenges posed by cybercrime. For instance, the United States and European Union have legislation in an effort to deal with cybercrime. ^2^

However, there is no study being done on how legislation was used to combat cybercrime through systematic literature review (SLRs). Combating cybercrime requires a range of ways including developing new legislation and regulation as well as awareness raising campaigns.

Thus, this paper discusses the systematic literature review of the use of legislation to combat cybercrime as SLRs can provide a valuable summarization of cybercrime legislation and allow for the identification of existing knowledge gaps and consequently, avenues for future research. In essence, this paper attempts to contribute to the current literature on cybercrime legislation in two ways; firstly by providing a thematically organised classification of the studies from the perspective of application of legislation, limitations, and recommendations and secondly the finding of this SLR can be used to propose a detailed framework to conduct a future research.

Three research questions are addressed in order to make these contributions:

1. What is the legislation to combat cybercrime in selected jurisdictions?
2. How is the legislation used to combat cybercrime in these jurisdictions?
3. What are the other approaches taken to combat cybercrime?

The objectives of this proposal are as follows:

1. To identify the legislation to combat cybercrime in selected jurisdictions.
2. To examine the legislation used to combat cybercrime in these jurisdictions.
3. To investigate the other approaches taken to combat cybercrime.

This SLR research paper is complementary to the existing research and attempts to provide the following contributions for those having an interest in cybercrime and cybercrime legislation to further their work.

**Table 1.** Research questions.

Research questions (RQ)	Discussion
RQ1: What are the legislation to combat Cybercrime in selected jurisdictions?	Different legislation has been used to combat Cybercrime. Understanding the impact of legislation will enhance the impact of legislation in combating cybercrime.
RQ2: How is the legislation used to combat in these jurisdictions?	Legislation can be utilised to resolve the problems of cybercrime the peculiar nature of cybercrime. This will provide an understanding of the importance of implementing an effective legislation in place.
RQ3: What are other approaches taken to combat Cybercrime?	Legislation is commonly used to control cybercrime. However, there are some other means taken in combating cybercrime. This question will look at different approaches taken in combating cybercrime.

**Figure 1.**
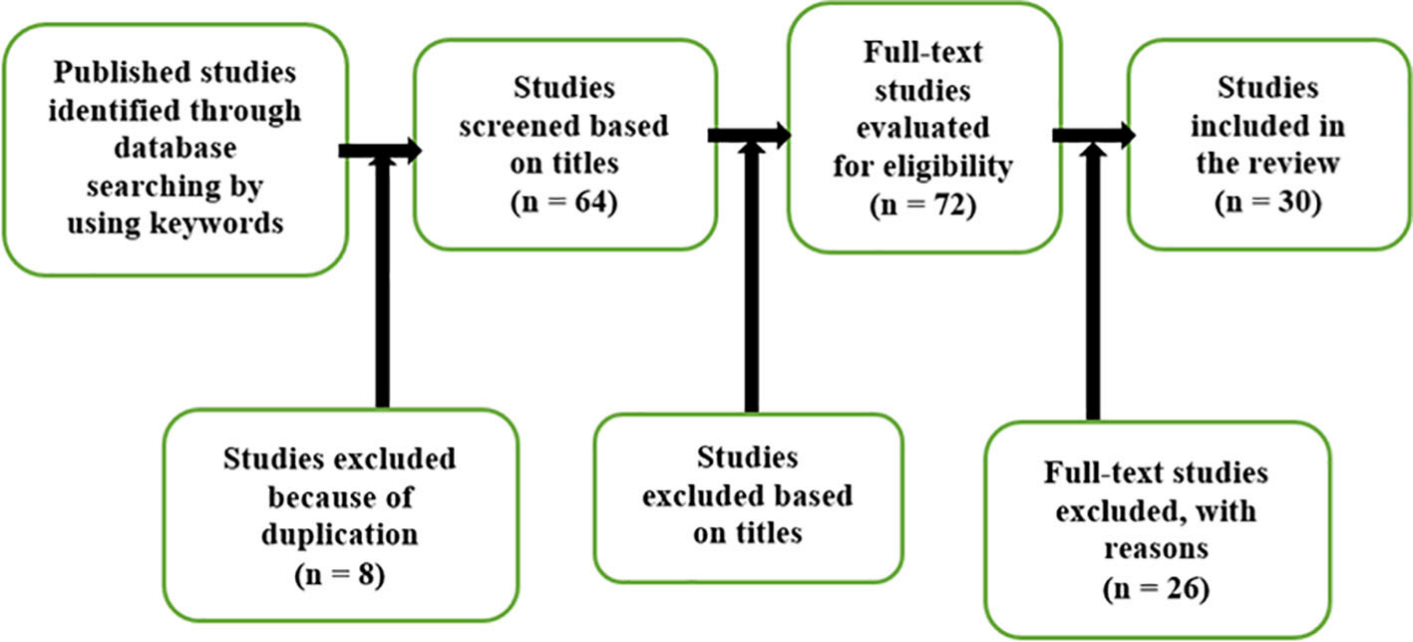
Attrition of papers through processing.

A total of 548 papers related to cybercrime legislation until the year 2021 were identified as initial studies ( Figure 1). Out of these papers, a total of 72 papers were further selected as primary studies for quality assessment to provide suitable benchmarks for comparative analysis towards related research. A comprehensive analysis was conducted on these 72 studies to present the ideas and considerations in the field of cybercrime legislation. A meta-analysis was conducted to improve the cybercrime legislation.

This paper analyses on how the international legal framework was used to combat cybercrime. The international legal framework can reduce the differences existing among the national laws as well as introducing new authorities and promoting international cooperation. The paper focuses on the necessity in international cooperation and implementation of international awareness as well as incorporating international norms into national legislation. The review study views cybercrime from a broader scope. All kinds of cybercrimes in general were looked into rather than a specific cybercrime such as cyber fraud. Different approaches were explored in raising cybercrime awareness and their effects, and investigating cyber legislation effectiveness along with the cybercrimes. No other review study is similar in work and scope. In essence, combating cybercrime requires a holistic understanding of the aspects involved and related interrelationships and there is a need to study the effectiveness of cybercrime legal framework. This article is based on an extensive analysis involving 72 papers relating to cybercrime legislation. This article contributes towards a comprehensive analysis on legal elements of cybercrime legislation and provides a summary of findings and the implications of this study for stakeholders to enhance the ability to successfully prosecute cybercrime offences.

The remainder of this paper followed on explanation of the methodology used for SLR in this paper and further on the discussion of the findings with continuation on a detailed discussion and reflection of the implications of the insights derived from the findings, then on the discussion of limitations and future recommendations on scope of research and finally 6 on concluding remarks.

## Methods

**Figure 2.**
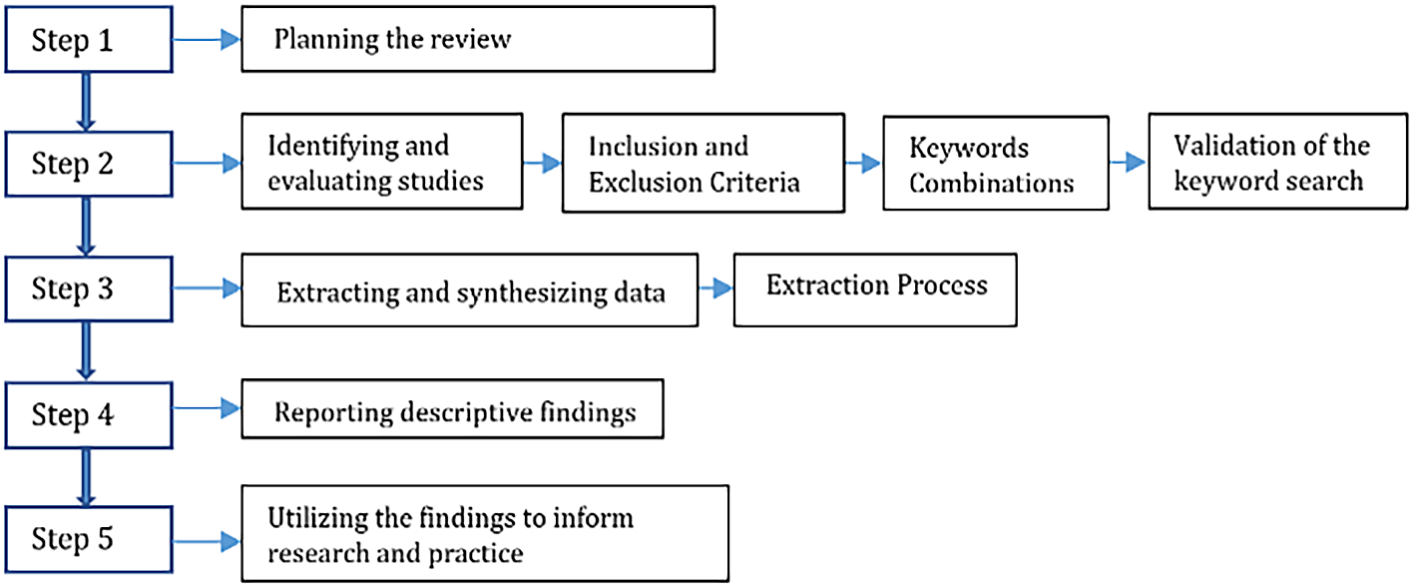
Structure of the method.

In order to meet the aims of answering the research questions, SLR analysis was conducted by applying several steps from the data collection to the synthesis of research findings. Figure 2 above shows the flowchart related to the stages of structure of the methods adopted for this SLR.

### Stage 1. Planning the review

The PRISMA analysis was applied including the suggested phases ^3^ during the data collection and data processing such as identification, screening, eligibility and included (see the detailed description in Table 2). The efficiency and effectiveness of the keyword search was investigated by the measurement of the relative number of articles which are relevant when the search was conducted by the additional keywords. If there is no increase in the relevant papers after adding the additional keywords, it is considered that the initial keyword used is valid. Once research questions and hypotheses are formulated, a topic modelling approach has been used to uncover the main topics which are then re-analyse to answer the research questions.

**Table 2.** Criterion on inclusion and exclusion.

Inclusion	Exclusion
• Papers written in English. • Papers discuss cybercrime legislation. • Papers discuss empirical studies related to the cybercrime legislation and application of legislation in combating cybercrime. • Information related to cybercrime or associated with the impact of legislation on cybercrime. • Peer-reviewed and published in journals listed in the reputable database.	• Papers written in other languages. • Papers do not discuss cybercrime legislation. • Papers relating to social, economic and political aspects of cybercrime. • Papers discussed on areas which are outside the scope of the study. • Other literatures such as blogs and outside the above stated searched database are not looked into.

### Stage 2. Identifying and evaluating studies

Primary study was done by using selected keywords to discover research trends of cybercrime legislation through systematic review from seven academic databases. To answer the research questions, keywords were selected by using AND and OR such as “cybercrime” OR “cyber-crime” OR “legislation” AND “combating”. The search platforms used were ACM Digital Library, Emerald, Hein Online, ProQuest, ScienceDirect, Scopus, and Westlaw Asia. The searches were conducted by using the keywords as stated above on these search backgrounds and filtered through the exclusion/inclusion criteria as stated in Table 2.

*Inclusion and exclusion criteria*

The empirical findings on case studies and cybercrime legislations and commentaries on the application and implementation of legislation in combating cybercrime are included in the studies. All the studies were written in English and peer-reviewed. The details of criterion for inclusion and exclusion can be seen in the table below.

The above criteria was adopted in order to select the papers to be used for this SLR analysis. To be selected in the study, the paper needs to fulfil all the criteria stated as inclusion and the paper will be excluded if it fulfils one of the excluded criteria.

*Keywords combination*

To identify the right set of key words and achieve the research objectives, seven electronic research databases such as ACM Digital Library, Emerald, Hein Online, ProQuest, ScienceDirect, Scopus and Westlaw Asia were searched by using the main keywords such as “cybercrime”, “cybercrime” AND “legislation”, “cybercrime” AND “legislation” AND “combating”, “cybercrime” AND “legislation” AND “cybersecurity”, and lastly “cybercrime” AND “legislation” AND “cybersecurity” AND “combating”. Searches were conducted until no new studies were identified within the scope of selected criteria

*Validation of keywords search*

A total of (72) studies were identified from the search of primary keywords in the databases as stated in Table 3. It was then reduced to (64) once duplicate studies were removed. By applying the inclusion/exclusion criteria, the papers were reduced to (56). All these (56) papers were analysed in detail and again the inclusion/exclusion method was applied and reduced to (46). These selected (46) papers were then reviewed in full and assessed for eligibility to be included in this SLR review. Further ^16^ papers were excluded for focusing on irrelevant issues and too general in nature. Finally, (30) papers were selected and used in this study.

**Table 3.** Database search summary.

		Keyword combinations					
No.	Online database	Cybercrime	cybercrime AND legislation	cybercrime AND legislation AND combating	cybercrime AND legislation AND cybersecurity	cybercrime AND legislation AND cybersecurity AND combating	(Selected papers)
1	ACM Digital Library	799	122	27	56	14	2
2	Emerald	972	337	169	82	43	11
3	Hein online	7642	4893	1796	1336	338	38
4	ProQuest	1473	775	195	54	29	10
5	Science Direct	3616	941	382	262	89	3
6	Scopus	1464	271	21	39	3	3
7	Westlaw Asia	2679	646	93	437	32	5
		18645	7685	2683	2266	548	72
	Total papers reviewed						72

### Stage 3. Extracting and synthesising data

All the selected papers then had their data extricated to evaluate the comprehensiveness of information to achieve the research objectives. The extraction process also provides the details of the studies for review and to be specific about how the paper can address the research question. It was done based on the selection process as discussed above. The data extracted are then categorised and then recorded in a worksheet.

Thematic synthesis was used as recommended by ^4^ to synthesize the results. An integrated approach to the synthesis process also was used to ensure that all the research objectives were achieved. The findings are described in the following section. Table 3 explains the number of papers at each phase of the process by the keywords search changes on each of the data platforms to focus on the final selection of papers.

### Findings

A substantial number of papers connected to cybercrime were identified based on the initial keyword searched. After going through the section process, only (30) papers were identified and analysed in detail. All the selected papers were read in full and relevant data were extracted and summarised in the table below. All primary studies focused on legislation relating to cybercrime in combating it.

### RQ1: What is the legislation to combat cybercrime?

Through the first research question (What is the legislation to combat cybercrime?) ( Table 1), it was aimed to identify the common legislation used to charge the cybercriminals. It is vital to note that this systematic literature review intends to focus on legislation used to combat cybercrime and no other type of legislation and thus the selection process focuses solely on the studies relating to cybercrime legislation. There are a number of studies regarding cybercrime in general and other aspects of it. However, studies on cybercrime legislation itself is very much limited.

There are a few international or regional instruments as stated in Table 5. The Convention on Cybercrime which is also known as Budapest Convention is the first and only binding international instrument on cybercrime and it also serves as a basic guideline for any country which is developing cybercrime related legislation to combat cybercrime. ^5^ However, not all the countries are a party or signatory to these conventions and harmonise with the international instruments ( Table 4). Although the Convention is under the auspices of the European Council, it is open to all the countries. The Convention focuses on Cybercrime & electronic evidence and provides a comprehensive, operational and functional solution for the investigation and prosecution of cybercrime both domestically and between Parties, with a global reach.

**Table 4.** Treaties & international agreements on cybercrime.

Name of instrument	Description	Legal status	
United Nations Convention Against Transnational Organized Crime (2000) (Palermo Convention)	The provisions of the treaty are highly relevant though it does not explicitly address cybercrime by compelling the parties to enact legislation for law enforcement cooperation and extradition.	Entry into force: 29 Sept. 2003. Signatories: 117 Parties: 178	
Convention on the Right of the Child (1989)	Article 34: State Parties to protect the child from all forms of sexual exploitation and abuse, including prostitution and pornography.	Entry into force: 2 Sept. 1990. Signatories: 140 Parties: 196	
Optional Protocol to the Convention on the Rights of the Child (2001)	Article 3(1) (c) prohibits child pornography and mentions Internet as a means of distribution.	Signatories: 121 Parties: 177	
Council of Europe Treaties Convention on Cybercrime (2001) (Budapest Convention)	The first international agreement to reduce cybercrime by harmonising with national laws. Improving investigative techniques and increasing international cooperation.	Signatories: 2 Ratification/accessions:66 (Non-EU countries: 22)	Non-EU countries: Argentina, Australia, Cabo Verde, Canada, Chile, Colombia, Costa Rica, Dominican Republic, Ghana, Israel, Japan, Mauritius, Morocco, Panama, Paraguay, Peru, Philippines, Senegal, South Africa, Sri Lanka, Tonga, United States of America
Additional Protocol to the Convention on Cybercrime, concerning the criminalisation of acts of a racist and xenophobic nature committed through computer systems (2003)	Extension of the Cybercrime Convention’s scope to cover offences of racist or xenophobic propaganda.	No. of signatures not followed by ratifications:12 No. of ratifications/accessions: 33 (non-EU member countries: 5)	Non-EU countries: Canada, Morocco, Paraguay, Senegal, South Africa.
Convention on the Protection of Children against Sexual Exploitation and Sexual Abuse (2007)	The Convention criminalises the solicitation of children for sexual purposes (“grooming”) and “sex tourism”.	No. of ratifications/accessions: 48 All the 47 countries who are the members of the Council of Europe and Tunisia are the only non-member.	
African Union Convention on Cyber Security and Personal Data Protection (data as on 18/06/2020)	The Convention was adopted in June 2014 and it addresses (i) electronic transactions, (ii) personal data protection, & (iii) cybersecurity and cybercrime.	Total countries: 55 Of signature: 14 Of ratification: 8 Of deposit: 8	The African Union (AU) consists of 55 countries.

**Table 5.** List of model laws.

Model title	Short form	Description	Jurisdiction
Model Law Computer and Computer Related Crime or Commonwealth Model Law	The Commonwealth	This is to assist the Commonwealth countries in enhancing the legal framework of cybercrime in criminalisation and investigation of computer and computer-related crimes.	Not widely adopted.
Cybercrime/e-crime: Model Policy Guidelines & Legislative Texts	HIPCAR	Synchronisation of ICT Legislation, Policies & Procedures.	Caribbean countries.
Pacific Island Regional Model Cybercrime Legislation	ICB4PAC	ITU-EC project Focus on capacity building and Legislative Framework.	Pacific Islands Countries.
Southern African Development Community (SADC) Model Law	HIPSSA	Focus on computer crime and cybercrime.	Sub-Saharan Africa region.
World Bank-OECS Model Law	OECS	Does not follow aby international best practice model. Criminalise social conduct on the internet.	Eastern Caribbean States (OECS)

In addition to these international instruments, there are Model cybercrime laws which propose to implement the best practice principles of substantive offences expressed by the Convention. Model Laws are usually developed by international organisations through the inter-governmental process of negotiations and formal procedure. Apart from the Commonwealth Model Law, the rest of the Model Laws did not go through the inter-governmental process. The table below explains the Model Laws currently implemented.

These ITU-EU led Models assisted the countries in Caribbean, Pacific and Sub-Sharan Africa region in drafting and adopting the legislation relating to cybercrime. ^6^ Their framework is based upon Commonwealth Model Law which is based upon the Budapest Convention. The common thing is to improve the legal framework of cybercrime legislation and create awareness on the importance of criminalising and adopting a specialised cybercrime law. In essence, these Models assist the countries in harmonising their legislation on cybercrime and attempt to redefine many aspects of cybercrime and jurisdictional matters. ^7^

The United States and the European Union criminal law are on the same path relating to criminal offences in cyberspace. ^8^ In fact, all countries should aim to pursue a common criminal policy in order to deter the cybercriminals effectively by coordinating in facilitating the detection of cybercrime, collecting the evidence, investigating and finally prosecuting the case successfully. Cybercrime has been on the rise due to the fact that there are loopholes in both national and international legislation that the existing legal framework cannot deter or effectively combat the rise of cybercrimes.

**Figure 3.**
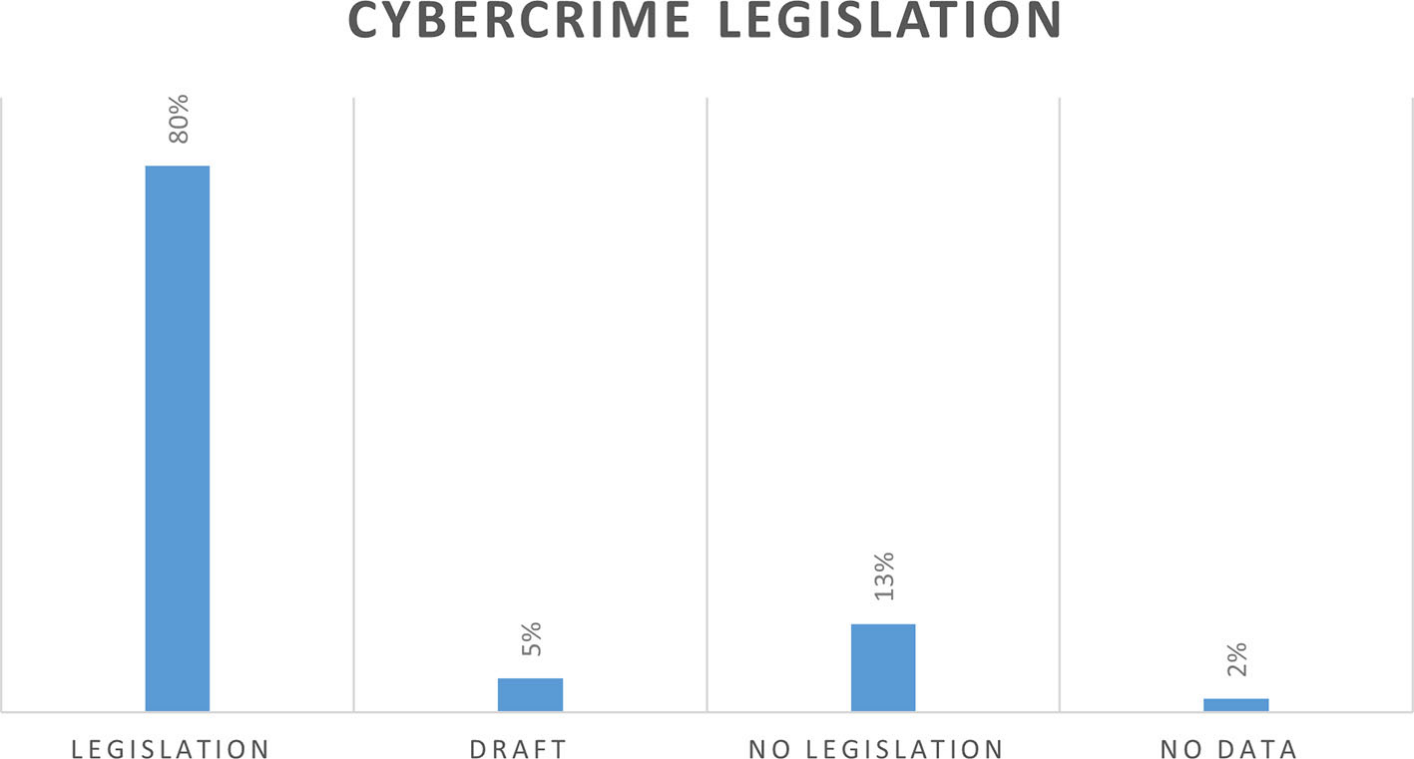
Percentage of countries with cybercrime legislation.

Based on the data retrieved from the United Nations Conference on Trade and Development, ^9^ a total of 154 countries have already enacted the cybercrime legislation whereas 13 percent of countries in the world do not have any legislation relating to cybercrime and 5% of countries have draft legislation. There was no data found on the two percent of countries ( Figure 3).

**Figure 4.**
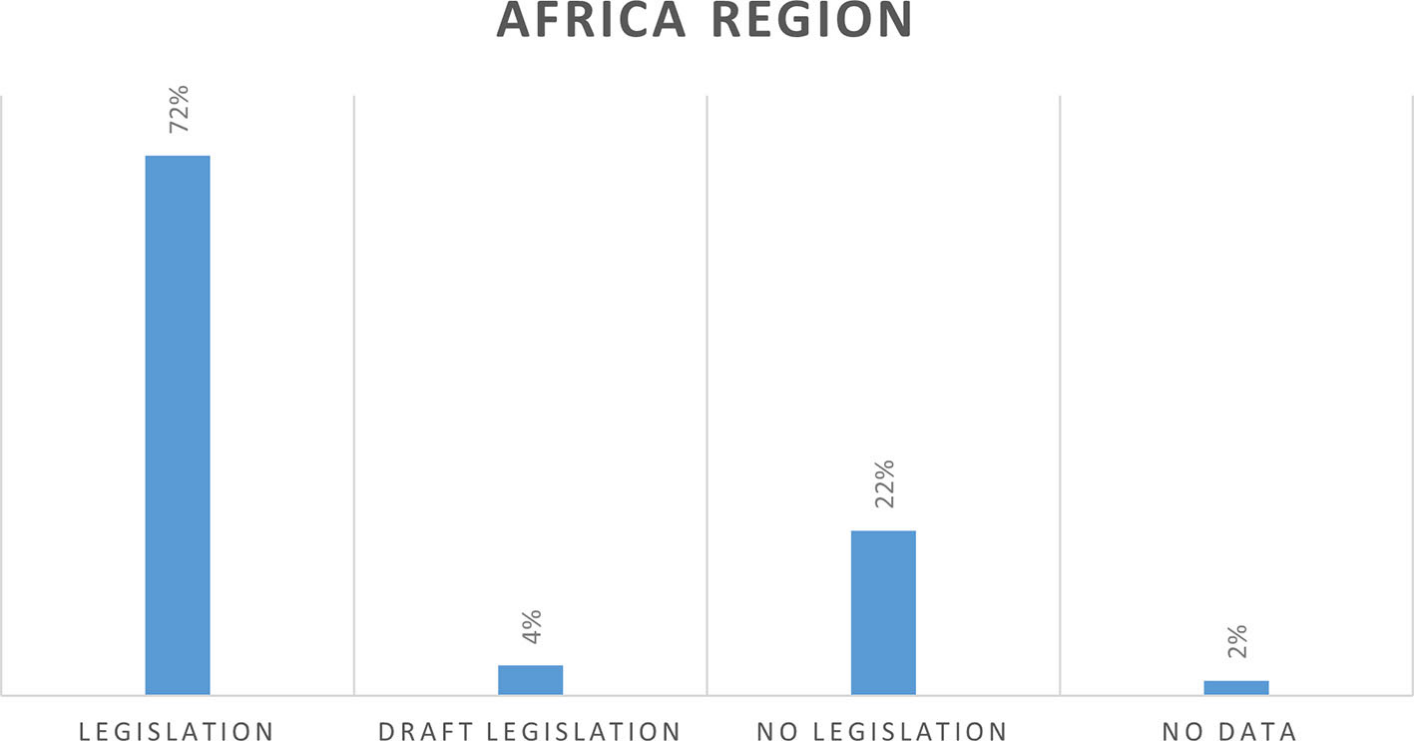
Cybercrime legislation in Africa region.

In the Africa region of 54 countries, 39 countries (72%) have cybercrime legislation, two countries (4%) have draft legislation, 12 countries (22%) do not have legislation and there is no data for one country (2%). (Siyanda, 2019) ( Figure 4).

**Figure 5.**
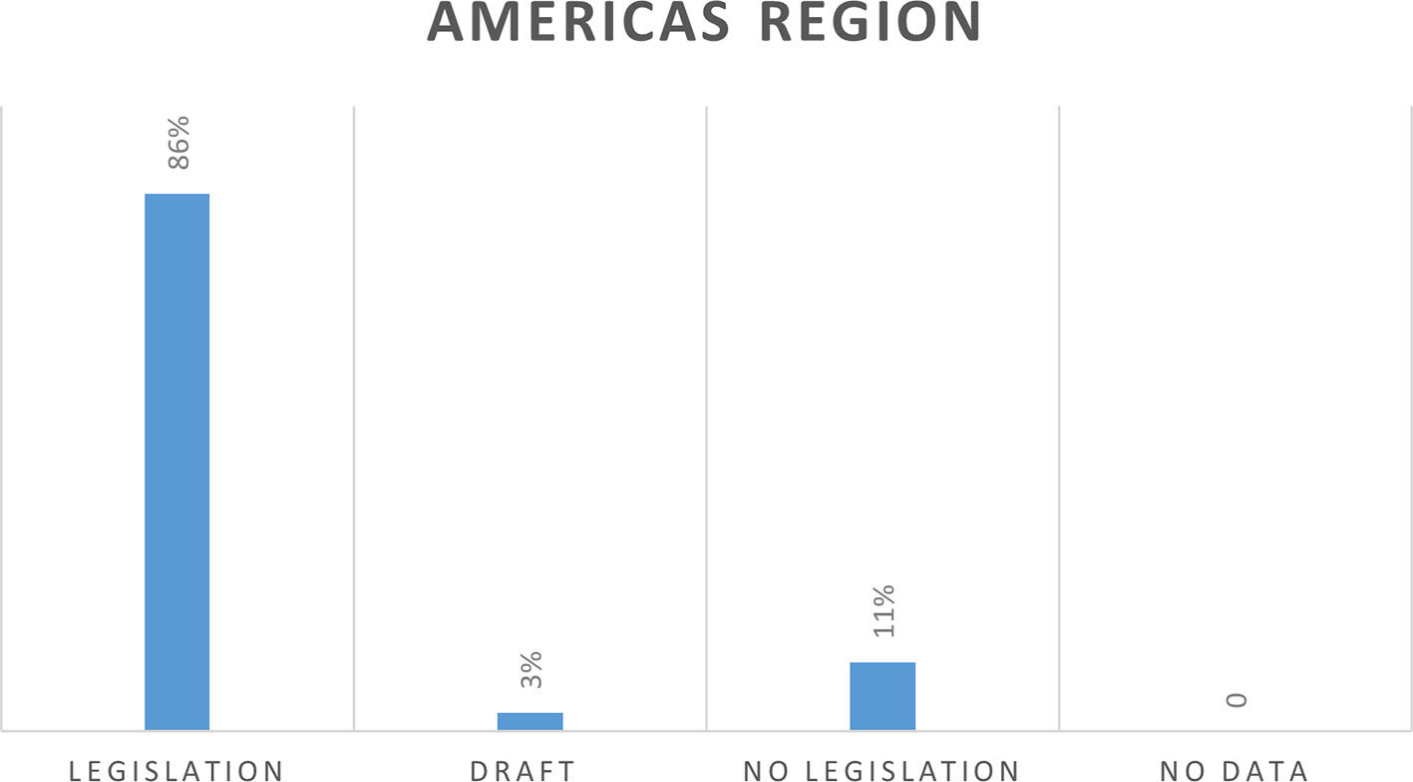
Cybercrime legislation in Americas region.

In the Americas which include 35 countries, 30 countries (86%) have cybercrime legislation, 1 country (3%) has draft legislation, and 4 countries (11%) do not have legislation ( Figure 5).

**Figure 6.**
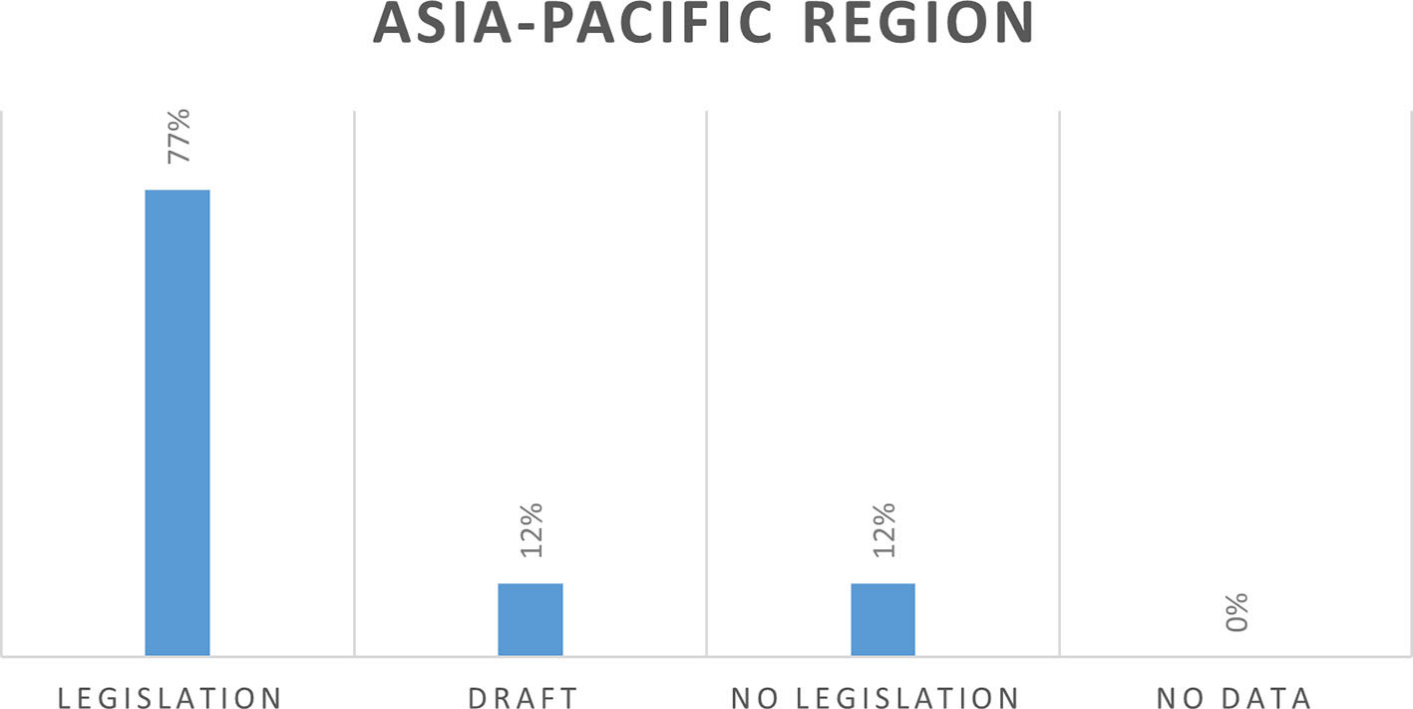
Cybercrime legislation in Asia-Pacific regio.

In the Asia Pacific region with 60 countries, 46 countries (77%) have legislation, seven countries (12%) have draft legislation, and seven countries (12%) do not have legislation ( Figure 6).

**Figure 7.**
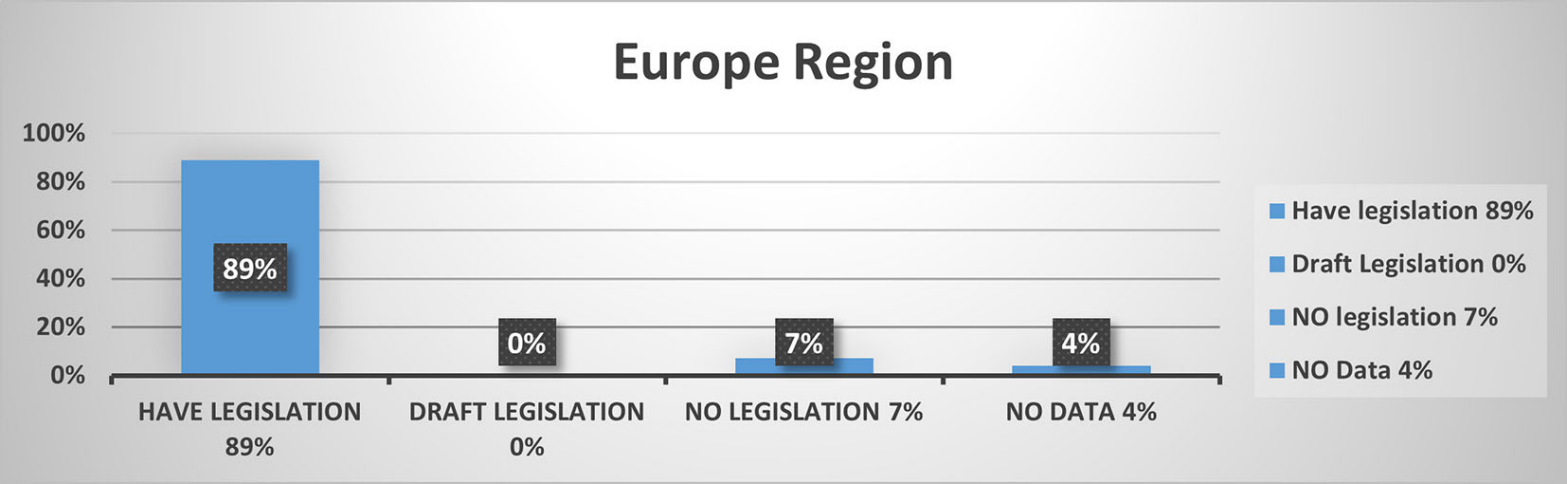
Cybercrime legislation in Europe region.

In Europe, out of 45 countries, 40 countries (89%) have legislation, three countries (7%) do not have legislation and no data for two countries (4%) ( Figure 7).

Even among the least developed countries, 31 countries (66%) have legislation, four countries (9%) have draft legislation and 12 countries (26%) have no legislation. Based on these statistics, it can be concluded that the majority of the countries in the world do have legislation relating to cybercrime. However, these legislations are obviously not efficient in deterring or combating the cybercrime effectively and the number of cybercrime cases have been on the rise constantly.

Different jurisdictions have used different types of legislation in charging/prosecuting the cybercriminals. ^10^ Some jurisdictions have specified cybercrime legislations and some had only relied on the legislation intended for traditional crimes. The table below shows an overview of the list of countries which have cybercrime legislation in English. Countries with the cybercrime bill, cybercrime draft legislation and cybercrime related legislation in non-English languages are excluded. It is essential to note that some jurisdictions used cybercrime as computer or digital crime. There are ways to improve the existing legislation relating to cybercrime and almost half of all published studies suggest harmonising national law with international law.

### RQ2: How is the legislation used to combat cybercrime?

Legislation plays a major role in combating cybercrime and empowering the authority in dealing with the challenges posed by cybercrime ( Table 6). Legislation involves both the national and international law relating to cybercrime. While traditional legislation focuses on physical objects, cybercrime is largely associated with data or information which are electronic in nature and thus traditional law cannot be used effectively in combating cybercrime. ^11^ Thus, national laws need specialised legislation to criminalise cybercrime.

**Table 6.** List of countries with legislation in relating to cybercrime.

No.	Name of the country	Title of the legislation
1	Australia	Criminal Code Act No. 12 of 1995 as amended in 2012, Cybercrime Legislation Amendment Act No. 120/2012
2	Brunei Darussalam	Computer Misuse Act, Revised in 2007
3	Canada	Criminal Code of 1985, Evidence Act 2010
4	China	Criminal Law as amended
5	Germany	Network Enforcement Act, 2017
6	Greece	Criminal Code and Law No. 4411/2016 - Ratification of the Convention on Cybercrime of the Council of Europe and the Additional Protocol to the Convention on Cybercrime, 2016
7	Hungary	Act C of 2012 on the Criminal Code
8	Iceland	General Penal Code
9	India	The Information Telecommunication Act of 2000, amended in 2008 - ITA
10	Indonesia	Law of the Republic of Indonesia Number 11 of 2008 Concerning Electronic Information and Transactions (in Indonesian and English)
11	Ireland	Criminal Justice (Theft and Fraud Offences) Act 2001, Criminal Damages Act 1991
12	Jamaica	Cybercrimes Act 2010, Criminal Justice Act 2014
13	Japan	Unauthorized Computer Access Law, 2013; Penal Code
14	Kenya	Kenya Information and Communication Act, 1998, The computer misuse and cybercrimes Act, 2018
15	Lao People's Democratic Republic	The Law on Prevention and Combating Cyber crime
16	Malawi	Electronic Transactions and Cyber Security Act 2016
17	Malaysia	Computer Crimes Act 1997
18	Mauritius	The Computer Misuse and Cybercrime Act (No. 22) 2003
19	Nepal	Electronic Transaction Act, 2063 (2008)
20	Netherlands	Telecommunication Act, 2014, Criminal Code 1881
21	New Zealand	Search and surveillance Act 2012; Film, Videos, and Publications Classifications Act 1993; Mutual Assistance in Criminal Matters Act 1992; Crime act 1961
22	Nigeria	Cybercrime Act 2015
23	Norway	Civil Penal Code 1902
24	Oman	Cyber Crime Law No. 12/2011 - Royal Decree
25	Pakistan	Prevention of Electronic Crime Ordinance 2009 Prevention of Electronic Crimes Act, 2016
26	Philippines	Cybercrime Prevention Act of 2012
27	Romania	Law No. 64/2004 - Ratification from the council of EU of the convention regarding cybercrime
28	Saudi Arabia	Anti-Cybercrime Law 1428/2007
29	Singapore	Cybersecurity Act No.9/2018
30	South Africa	Electronic Transactions and Communication Act 2002
31	South Sudan	Penal Code Act, 2008 (in English)
32	Sri Lanka	Payment Devices Frauds Act 2006, Computer Crime Act No 24 2007
33	Sudan	Law No. 14 on Information Technology Crime; Cyber Crimes Act 2007
34	Switzerland	Criminal Code 1937, as amended in Feb 2020
35	Thailand	Computer Crime Act 2007
36	Uganda	Computer Misuse Act, 2011
37	United Arab Emirates	Federal Decree-Law no. (5) of 2012 ON COMBATING CYBERCRIMES
38	United Kingdom of Great Britain and Northern Ireland	The Computer Misuse Act of 1990, as amended in 2013
39	United Republic of Tanzania	Cybercrime Act, 2015
40	United States of America	Title 18 - Crimes and Criminal Procedure Computer Fraud and Abuse Act 198
41	Viet Nam	Law on cyber information security, 2015; Law on network information security, 2015
42	Zambia	The Electronic Communication and Transactions Act 21 (ECT Act) 2009

**Figure 8.**
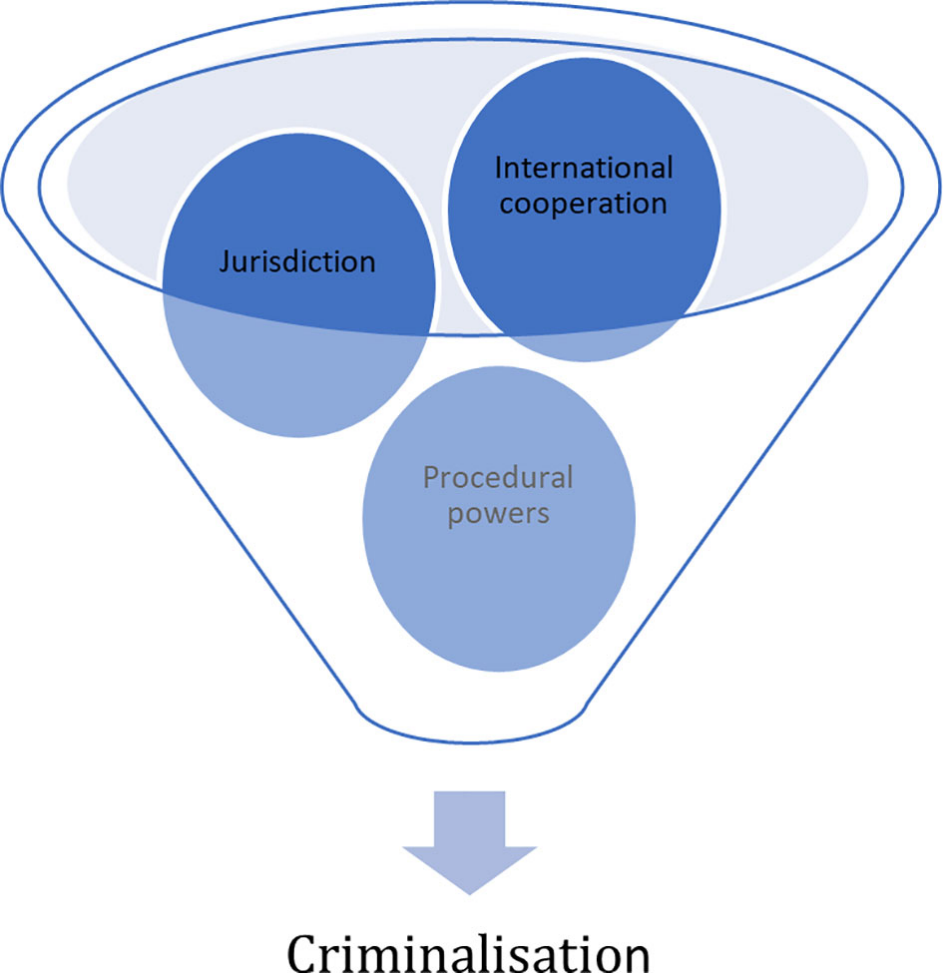
Essential Instruments Involved in Combating Cybercrime.

With the exception of spam offences, most of the jurisdictions in some western and European countries have criminalised and imposed criminal penalty on cybercrime cases such as misusing of computer tools and towards racism, xenophobia and online solicitation or exploitation of children. ^12^ However, the penalties imposed are not strong enough in deterring the cybercriminals from committing the crimes in cyberspace that there is a constant rising of cybercrime cases around the world. ^13^ Cybercrimes relating to integrity, confidentiality and accessibility of computer systems are mostly regarded as specific cyber offenses in many jurisdictions, whereas offences such as fraud, breach of privacy and identity offences are regarded as general cyber offences. ^14^ Those countries which impose criminal penalties or criminalise cybercrime use different methodology in application such as Finland regulates cyber offences via the new chapters of penal code. The evolving nature of cybercrime cannot be controlled efficiently unless the legislation is updated enough to be used to investigate, prosecute and adjudicate the cybercrime offenders. The cooperation from the internet service providers and internet industry players also play a crucial part in order for the law enforcement officers to detect, prevent and respond to the cybercrimes. Thus it is material that effective and updated legislation in par with the rapid advancement of technology must be in place in order to combat ever rising cybercrime globally. With this view in mind, the United States House of Representative Tom Graves sponsored a bill named Active Cyber Defense Certainty (“ACDC”) Act to update the Computer Fraud and Abuse Act under which the organisations can take active measures to go beyond the boundaries of their own networks. Procedural laws should also be enhanced or amended to ensure that issues related to cybercrime such as digital evidences are to be easily collected, stored and presented at the Court without any difficulty like any other traditional crimes. ^15^ Urgent International collaboration is needed to take every possible approach to criminalise all types of cybercrimes and to adopt specific procedural rules and investigation powers to investigate and prosecute cybercrimes specifically ( Figure 8). ^16^

In the United Kingdom, the Computer Misuse Act 1990 which has been amended several times imposes criminal sanctions for cybercrime. Also under the Serious Crime Act 2015, criminal penalties can be imposed for an unauthorised act of causing serious damage to security or to the economy of the country. Even though the purpose is to punish and deter the criminals from committing the cybercrime, it does not deter the cybercriminals effectively since there still is a constant rise in cybercrime cases. ^17^

Thus, to use legislation as an effective tool to combat cybercrime, cybercrime legislation must cover an extensive list of offences committed in cyberspace like some jurisdictions focus only on selective cybercrimes such as child pornography and child protection as criminal offences. ^18^ Only a limited number of jurisdictions address and impose the duty on the service providers to monitor and voluntary supply of information as well as taking down the notifications and liability of access. Eighty percent of European countries have criminalised the cybercrimes compared to the other parts of the world whereas approximately only sixty percent of these countries criminalise the cybercrimes. ^19^^,^^20^

### RQ3: What are the other approaches taken to combat cybercrime?

In addition to the enhancement of existing legislation, there is also a necessity to focus on the development of methodological manuals on the investigation of cyber related crimes in law enforcement. A substantial number of primary studies suggest that public awareness, technical approaches and ethical online education are as important as having efficient legislation in combating cybercrime. There is also a need for the international community to come together as one united nation to voice and be united in combating this cybercrime.

Even with the best possible legal framework, to address the technical vulnerabilities, private sectors must also contribute in fighting the rise of cybercrime by addressing technological vulnerabilities in the system and collaborating in operating the system with other jurisdictions. With the rapid development of technology, it is imperative to prevent and combat cybercrime by both public and private sectors. The role played by the criminal justice system is also important and thus have to ensure that justice officers are well equipped with the latest technology development. Thus, it is important to have cybercrime strategies in order to meet the challenges of law enforcement and prosecution. Raising public awareness of cybercrime, cooperation of private-public partnerships, criminal law enforcement and justice capacity are the areas that should be addressed as the cybercrime strategies. The importance of raising public awareness of cybercrime was highlighted in the United Nations Guidelines for Prevention of Crime. Most jurisdictions are aware of the importance of raising public awareness of cybercrime and engage in awareness campaigns. ^21^^,^^22^

## Discussion

Efforts have been made in all the jurisdictions to amend the existing legislation to address the need for higher standards of law enforcement. However, due to the nature of cybercrime, it is difficult to determine the jurisdiction of the court in practice. Firstly, it is not restricted to a particular country or territory. It can happen in any part of the world and involve multiple jurisdictions. Secondly, cybercrime can take place in any jurisdiction and it is not possible to exclude the jurisdiction of the states even though the legislation of that particular state is very weak. For example, if malicious cyber activities have been committed by using the cyber infrastructure of the country whose cyber legislation is very weak and attack on the other states, it is none to impossible to prosecute the cybercriminal in that particular jurisdiction.

It should be noted that preventing the crime is always better than waiting for it to happen since it effectively reduces the damages suffered as a result of the crime. ^23^ Cybercrime is complex and prevention of it generally involves a high degree of collaboration both at national and international levels between private and public sectors. ^24^ Technologies and networks are crucial infrastructure in preventing crime. ^25^ At the moment, there is no definite figure of losses suffered by cybercrimes though it is obvious that billions of damages have occurred every year. The availability of cyber security insurance may mitigate the losses suffered as a result of cybercrime. No doubt, there is a serious need to promote the implementation of precautionary actions in all the jurisdictions. ^26^ It has to be acknowledged that there are some limitations on this study since the findings were interpreted based on the articles written in English only. Some other important points may have been found if the research was extended to cover other languages. Also there may be some other articles which were not listed in the data cases that these studies have based upon. However, the findings of this research can be concluded as a reasonable representation of current and latest literature available and could serve as a pertinent reference to the policymakers.

## Future research and recommendation

It is important to have adequate and efficient responses in place in order to combat cybercrime efficiently. Because of the nature of cybercrime which is borderless in nature, it is pertinent that there is a harmonisation of national law with the international law as well as a strong cooperation among the states. One of the most important factors is to criminalise the computer related offenses and having a special cybercrime legislation to govern these types of crime specifically and not to just merely rely on the traditional crime legislation. Cybercrime legislation must clearly spell out the cyber offences to ensure that cybercrime offenders can be prosecuted successfully.

Analysis of United Nation documents show that integrity, confidentiality and accessibility of computer systems are criminalised as core specific cyber offences in many countries whereas fraud or forgery, breach of privacy and identity offences are more often criminalised as general cyber offences. At the same time, sufficient procedural powers must be given to investigate the cybercrime efficiently. There should be proper steps to ensure key evidence is properly kept to prove the crime. Challenges faced by law enforcement to prosecute the cybercrimes have to be addressed. However, it is not sufficient. Need to criminalise all types of cybercrime and urgently need to fill the gap in the existing legislation by strengthening the legal response in facing the challenges of cybercrime.

Cybercrime in general involves multi-jurisdictional issues and thus combating cybercrime is bound to a number of challenges both to public and private sector; public sector issues involve legislation, investigation and prosecution whereas private sector issues involve technical vulnerabilities of the system in its design and operational aspects. In essence, there has to be specialised enforcement in investigation and prosecution involving cybercrimes due to the nature of electronic evidence involved which is different from traditional crimes. Resources should be centralised in one place in order to build capacity on specialised investigation and analysing the electronic evidence. At the same time, law enforcement authorities have to be trained consistently to ensure that they are abreast with all the latest technology.

## Conclusion

Many countries have imposed criminal sanctions on crimes related to internet related crimes in order words called as cybercrimes as a legal response. Based on the INTERPOL’s ASEAN cyber threat assessment in January 2021, it was predicted that the growing trend in cybercrime is expected to continue since cybercriminals are well-organised and share resources and knowledge to their benefit. Thus, it is essential that there is a collaboration among the law enforcement agencies across the region. Since cybercrime is evolving constantly, it is vital for all the countries to enhance their legislation as well as the responses to the ever-evolving cybercrime threat in the digital era of the century. It is increasingly important to harmonise cybercrime legislation which cannot be achieved without the cooperation of the international operation. Those countries which do not criminalise cybercrime need to do so on an urgent basis by either enacting new legislation or amend the existing legislation and incorporate the provisions relating to cybercrime. Aggravated punishment should be meted out ensuring that effective penalties are imposed on cybercrime offenders. Public awareness and education programmes should be enhanced.

There are divergent national approaches globally because of the dissimilarities in the legal system historically and socio-cultural backgrounds. Thus, it is essential to have a truly effective legal response by cooperating among the international communities by coming up with an international legislation endorsed by industry executives, government officials, security professionals both from public and private sectors to come together and at the same time increase the much needed public awareness of the cybercrime. In essence, there is a need to have a bigger and better international cooperation on evidence collection, information sharing and criminal prosecution of those involved in cybercrimes. However, all these could not be done unless all the nations to begin with must have substantive provisions in the respective legislation which criminalise the cybercrimes. There are more questions than answers based on analysis of legal framework in combating cybercrime and it is indisputable that the legal framework of cybercrime has to be constantly adjusted and evolved to the extremely fast and highly complicated technical challenges.

## Data availability

Figshare. Data_Excelfile.xlsx DOI: https://doi.org/10.6084/m9.figshare.20097614.v1. ^27^ This project contains the following underlying data:

- The data from the selected publications were extracted to assess the comprehensiveness and meet the study objectives which have been subsequently classified and entered into a spreadsheet.

Figshare: PRISMA checklist: A systematic literature review on cybercrime legislation. DOI: https://doi.org/10.6084/m9.figshare.20218730.v1

Figshare: Data_Excelfile

DOI: https://doi.org/10.6084/m9.figshare.20097614.v1

Data are available under the terms of the Creative Commons Attribution 4.0 International license (CC-BY 4.0).

## Author contributions

1. Shereen Khan: Conceptualization, Formal Analysis, Investigation, Methodology, Validation, Visualization, and Writing – Original Draft Preparation
2. Tajneen Affnaan Saleh: Writing – Review & Editing
3. Magiswary Dorasamy: Conceptualization, Project Administration, Validation, Visualization, and Review
4. Nasreen Khan: Investigation, and Methodology
5. Olivia Tan Swee Leng: Conceptualization, and Methodology
6. Rossanne Gale Vergara: Data Curation, and Resources

## References

[1] Bechara FR Schuch SB : Cybersecurity and Global Regulatory Challenges. *Journal of Financial Crime.* 2021;28(2):359–374. 10.1108/JFC-07-2020-0149

[2] Redford M Jefferson T : U.S. and EU Legislation on Cybercrime. *European Intelligence and Security Informatics Conference.* 2011;34–37. 10.1109/EISIC.2011.38

[3] Moher D Liberati A Tetzlaff J : Preferred reporting items for systematic reviews and meta-analyses: the PRISMA statement. *PLoS Medicine.* 2009;6(7):e1000097. 10.1371/journal.pmed.1000097 19621072 PMC2707599

[4] Cruzes DS Dyba T : Recommended steps for thematic synthesis in software engineering. *International Symposium on Empirical Software Engineering and Measurement.* 2011;275–284.

[5] Clough J : A World of Difference: The Budapest Convention on Cybercrime and the Challenges of Harmonisation. *Monash University Law Review.* 2013;40(3):698–736.

[6] Vertes-Olteanu A : Evolution of the criminal legal frameworks for preventing and combating cybercrime. *Journal of Eastern-European Criminal Law.* 2014;1:84–96.

[7] Vorobets K : The main directions of cybercrime prevention in Ukraine. *European Journal of Law and Public Administration.* 2019;6(1):74–88. 10.18662/eljpa/65

[8] United Nations Office on Drugs and Crime (UNODC): *Comprehensive Study on Cybercrime.* New York: United Nations;2013. Reference Source

[9] United Nations Conference on Trade and Development: *Cybercrime Legislation Worldwide, United Nations, Editor.* New York: United Nations Publication;2018.

[10] Sviatun O Goncharuk O Roman C : Combating Cybercrime: Economic and Legal Aspects. *WSEAS Trans. Bus. Econ.* 2021;18(18):751–762. 10.37394/23207.2021.18.72

[11] Duryana Binti M : Combating the threats of cybercrimes in Malaysia: The efforts, the cyberlaws and the traditional laws. *Computer Law & Security Review.* 2013;29(1):66–76. 10.1016/j.clsr.2012.11.005

[12] Christou G : The challenges of cybercrime governance in the European Union. *European Politics and Society.* 2018;19(3):355–375. 10.1080/23745118.2018.1430722

[13] Cassim FF : Protecting personal information in the era of identity theft: Just how safe is our personal information from identity thieves. *Potchefstroom Electronic Law Journal.* 2015;18(2):68–110. 10.4314/pelj.v18i2.02

[14] Correia SG : Responding to victimisation in a digital world: a case study of fraud and computer misuse reported in Wales. *Crime Science.* 2019;8(4). 10.1186/s40163-019-0099-7

[15] Giuseppe C Damian AT Willem-Jan Van Den H : Cybercrime threat intelligence: A systematic multi-vocal literature review. *Computers & Security.* 2021;105:102258. 10.1016/j.cose.2021.102258

[16] Chigada J Madzinga R : Cyberattacks and threats during Covid-19: A Systematic Literature Review. *South African Journal of Information Management.* 2021;23(1):a1277.

[17] Catherine F Marek P : Fighting Cybercrime: A Review of the Irish Experience. *International Journal of Cyber Criminology.* 2020;14(2):383–399. 10.5281/zenodo.4766528

[18] Quayyum F Cruzes DS Jaccheri L : Cybersecurity awareness for children: A systematic literature review. *International Journal of Child-Computer Interaction.* 2021;30:100343. 10.1016/j.ijcci.2021.100343

[19] Calderoni F : The European legal framework on cybercrime: striving for an effective implementation. *Crime, Law and Social Change.* 2010;54(5):339–357. 10.1007/s10611-010-9261-6

[20] Buono L : Gearing up the fight against cybercrime in the European Union: new set of rules and the establishment of the European cybercrime centre (ec3). *New Journal of European Criminal Law.* 2012;3(3-4):332–343. 10.1177/203228441200300307

[21] Aloul FA : The need for effective awareness. *Journal of Advances.* 2012;3(3):176–183. 10.4304/jait.3.3.176-183

[22] Catherine F Lorraine BG Jennifer K : Fighting Cybercrime: A Review of the Irish Experience. *International Journal of Cyber Criminology.* 2021;14(2).

[23] Majid Y Jacqueline D : Image-Based abuse, Non-consensual Pornography, Revenge Porn: A Study of Criminalization and Crime Prevention in Australia and England & Wales. *International Journal of Cyber Criminology.* 2019;13(2):578–594.

[24] Mohammed SA : Global Surge in Cybercrimes – Indian Response and Empirical Evidence on Need for a Robust Crime Prevention System. *International Journal of Cyber Criminology.* 2020;14(2).

[25] Jayasekara SD Abeysekara I : Digital forensics and evolving cyber law: case of BIMSTEC countries. *Journal of Money Laundering Control.* 2019;22(4):744–752. 10.1108/JMLC-02-2019-0019

[26] Moise A : Modernization of Romanian Legislation on Preventing and Combating Cybercrime and Implementation gap at European level. *Revista de Stiinte Politice.* 2015;45:186–199.

[27] Khan S Affnaan T Dorasamy M : Data_Excelfile.xlsx. figshare. Dataset. 2022. 10.6084/m9.figshare.20097614.v1

