# Supplementary material for: A systematic literature review on cybercrime legislation
Source: F1000Res. 2022 Aug 23;11:971. [Version 1] doi: 10.12688/f1000research.123098.1 (PMC11384205; doi:10.12688/f1000research.123098.1)
Supplement: Peer review history

# Reviewer report: Reviewer response for version 1

**DOI:** 10.5256/f1000research.135168.r308598

**Published:** 2024-09-09

**Authors:** Duryana Mohamed

**Affiliations:** 1 International Islamic University Malaysia, Kuala Lumpur, Federal Territory of Kuala Lumpur, Malaysia

I found this research very interesting and new to me. As someone from a legal background and with some knowledge on cybercrime and Cyberlaws, I usually study and analyze the problem based on laws and cases then comment on whether the regulatory frameworks are adequate or not. Sometimes, I do a comparative analysis with other common law jurisdictions.

 This research focuses on the SLR of cybercrime legislations of three regions which I think are quite comprehensive though they cover only English versions. 

 Overall, they can be considered as acceptable as I have not seen this kind of research before. Nevertheless, I found the findings are quite common and mostly similar to what have or had been recommended by most authors who wrote on regulatory frameworks of Cybercrimes, their adequacy, or their effectiveness.

Are the rationale for, and objectives of, the Systematic Review clearly stated?

Yes

Is the statistical analysis and its interpretation appropriate?

Yes

If this is a Living Systematic Review, is the ‘living’ method appropriate and is the search schedule clearly defined and justified? (‘Living Systematic Review’ or a variation of this term should be included in the title.)

Yes

Are sufficient details of the methods and analysis provided to allow replication by others?

Yes

Are the conclusions drawn adequately supported by the results presented in the review?

Yes

Reviewer Expertise:

cybercrime, cyber laws, professional ethics and electronic evidence.

I confirm that I have read this submission and believe that I have an appropriate level of expertise to confirm that it is of an acceptable scientific standard.

# Reviewer report: Reviewer response for version 1

**DOI:** 10.5256/f1000research.135168.r148308

**Published:** 2022-09-06

**Authors:** Sagwadi Mabunda

**Affiliations:** 1 University of the Western Cape, Cape Town, South Africa

This article begins with an overview of the rise of cybercrime since the advent of the pandemic, detailing the losses encountered. It rightly expresses a lack of studies cataloging cybercrime legislation and regulations. The paper seeks to do two things: 1. provide thematically organised classification of legislation etc. through a systematic literature review (SLR) and 2. using the outcome of the SLR to propose a detailed framework to conduct future research. 

 The authors have identified appropriate research questions which are in line with the objectives they seek to achieve. Further, they have adequately defined their methodology by identifying the keywords employed and the sources consulted. There also appears to be a substantial number of sources consulted which makes for an expectation of credible results. The authors have identified the legislation, policies, conventions and model laws expected to be included in a study such as this. The analysis is not limited a particular region, but rather takes a global approach from which the reader can use as a starting point for their own research. The graphs and tables provided are clear and easy to comprehend. 

 The authors also provided insight into the substance of the legislation such as how jurisdictions criminalise and prosecute some cybercrimes. 

 While not fatal to the quality of the paper, it would benefitted from a more robust discussion section. Of the two things sought to be done by the authors, I am of the view that no.1 was successfully executed but no.2 could benefit for more exploration. The information contained in the paper makes a good contribution to the literature and as such is deserving of being indexed.

Are the rationale for, and objectives of, the Systematic Review clearly stated?

Yes

Is the statistical analysis and its interpretation appropriate?

Yes

Are sufficient details of the methods and analysis provided to allow replication by others?

Yes

Are the conclusions drawn adequately supported by the results presented in the review?

Yes

Reviewer Expertise:

Cybercrime, criminal law, anti-money laundering, & anti-corruption law.

I confirm that I have read this submission and believe that I have an appropriate level of expertise to confirm that it is of an acceptable scientific standard.
